# Up-Conversion Sensing of 2D Spatially-Modulated Infrared Information-Carrying Beams with Si-Based Cameras

**DOI:** 10.3390/s20123610

**Published:** 2020-06-26

**Authors:** Adrián J. Torregrosa, Emir Karamehmedović, Haroldo Maestre, María Luisa Rico, Juan Capmany

**Affiliations:** 1 Communications Engineering Department and I3E, Universidad Miguel Hernández, Avda. de la Universidad s/n, 03202 Elche, Spain; hmaestre@umh.es (H.M.); jcapmany@umh.es (J.C.); 2 Faculty of Engineering and Natural Sciences, International University of Sarajevo, Hrasnička cesta 15, 71210 Sarajevo, Bosnia and Herzegovina; ekaramehmedovic@ius.edu.ba; 3 Computer Science Department, Universidad de Alicante, Ctra. San Vicente s/n, 03690 San Vicente del Raspeig, Alicante, Spain; rico@dtic.ua.es

**Keywords:** image up-conversion, infrared imaging, free-space laser communications, intra-cavity wavelength conversion, infrared sensing

## Abstract

Up-conversion sensing based on optical heterodyning of an IR (infrared) image with a local oscillator laser wave in a nonlinear optical sum-frequency mixing (SFM) process is a practical solution to circumvent some limitations of IR image sensors in terms of signal-to-noise ratio, speed, resolution, or cooling needs in some demanding applications. In this way, the spectral content of an IR image can become spectrally shifted to the visible/near infrared (VIS/NWIR) and then detected with silicon focal plane arrayed sensors (Si-FPA), such as CCD/CMOS (charge-coupled and complementary metal-oxide-semiconductor devices). This work is an extension of a previous study where we recently introduced this technique in the context of optical communications, in particular in FSOC (free-space optical communications). Herein, we present an image up-conversion system based on a 1064 nm Nd^3+^: YVO_4_ solid-state laser with a KTP (potassium titanyl phosphate) nonlinear crystal located intra-cavity where a laser beam at 1550 nm 2D spatially-modulated with a binary Quick Response (QR) code is mixed, giving an up-converted code image at 631 nm that is detected with an Si-based camera. The underlying technology allows for the extension of other IR spectral allocations, construction of compact receivers at low cost, and provides a natural way for increased protection against eavesdropping.

## 1. Introduction

Recently, a pioneering application was presented where a 2D Quick Response (QR) code was embedded in an eye-safe IR (infrared) laser beam at 1550 nm, transmitted in space, successfully read by the silicon focal plane arrayed (Si-FPA) image sensor of a receiving smartphone, and correctly interpreted by the smartphone software [1]. To excite the Si sensor, the IR laser beam went previously through a real-time image frequency up-conversion process, based on nonlinear optics heterodyning in a sum-frequency mixing process, where a 1064 nm local oscillator laser shifted the IR image spectrum to around 631 nm (contained within the efficient spectral detection region of Si), while preserving its 2D spatial information with great fidelity.

The availability of IR imaging systems providing information for interpretation and analysis in spectral regions where the human eye or cameras in the visible are not sensitive, has allowed great progress in basic research and technologic development. In this context, active imaging systems are used for collecting information resulting from illumination of targets at specific wavelengths. Such systems are widely used for detection and ranging. They have been also proposed for determination of object characteristics such as temperature, shape (2D and 3D), texture, composition, and other features by non-invasive or non-damaging inspection (i.e., in vivo cells or tissues or pigments) [2,3,4,5]. They are also used for visualization through scattering media like mist, fog, or smoke [6,7]. At present, these techniques are commonly applied in diverse fields such as in biomedicine [8], surveillance, defense [9], food control [10], pests/crops monitoring [11], cultural heritage [12], machine vision systems [13], and more. Furthermore, active imaging in short-wave IR (SWIR) and mid-wave IR (MWIR) wavelengths would benefit from eye-safe (1.55-μm band) and low attenuation windows (1.55-μm, 2-μm, and 3–5-μm bands), being particularly suited for remote sensing applications [14]. The advantages of systems operating directly in these bands have not been fully exploited yet, due to the limitations posed by the available IR sensor technology. Either thermal-based or with quantum semiconductor-based devices (such as PbS, PbSe, HgCdTe, InSb, or InGaAs), cannot compete with Si sensors in terms of resolution, sensitivity, signal to noise ratio, dynamic range, speed, cost, or size due to the need of cooling sub-systems to reduce noise at room temperature [15]. Thus, despite the availability of image sensors for direct detection across the whole infrared spectrum, optical frequency up-conversion detection based on nonlinear sum-frequency mixing (SFM) of an IR signal with a local oscillator laser wave in a nonlinear crystal and subsequent detection with Si VIS/NIR sensors, can provide advantages in some specific cases. In particular, in applications that may require uncooled detector operation, lower price, better signal-to-noise (S/N) ratio, higher speed, or better resolution than that provided by IR sensors.

A few years after the discovery of nonlinear optical parametric interactions with laser beams [16,17], Midwinter and Warner realized that the up-conversion process based on SFM could help in overcoming some IR detector limitations [18]. The SFM process itself was shown to be theoretically noiseless with the overall S/N characteristics of the full detection system essentially inheriting those of the detector alone. These S/N characteristics are superior in case of Si-based sensors as compared with other uncooled IR semiconductor detectors. Thus, shifting the signal spectral region to that detectable by VIS/NIR detectors could have some benefits in particular situations. Cooling IR sensors to improve the S/N always represents an increase in cost and a complexity in high performance IR semiconductor detectors. It becomes apparently clear that in up-conversion detection the S/N ratio improves with up-conversion efficiency due to the larger signal power available. This was verified soon for point detectors, i.e., detectors that integrate the full energy or power received within their surface. Presently, enhancement factors of 64 in sensitivity have been reported in up-conversion detection with uncooled Si with respect to direct InSb detection around 3.2 µm in the MIR [19].

Soon after, J.E. Midwinter realized the possibility of full image up-conversions [20]. Due to poor up-conversion efficiency achieved at that early stage, the topic received little research interest until a decade ago, when the combination of an intra-cavity image up-conversion process and the use of poled nonlinear ferroelectric crystals of high nonlinear effective coefficient boosted image up-conversion efficiency, particularly in case of continuous-wave (CW) systems [21]. A review of present state of the art and recent progress can be found in [22].

**Figure 1:**
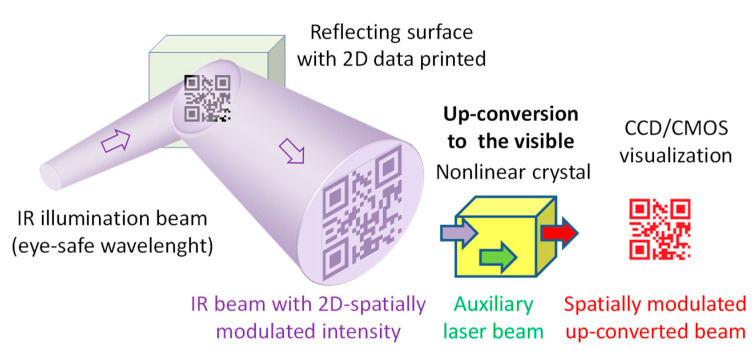
Remote sensing by up-conversion of encoded 2D data from IR (infrared) illumination. CCD/CMOS: charge-coupled and complementary metal-oxide-semiconductor devices.

This work is an extension of a previous study [1], where we introduced image up-conversion systems in the context of optical communications, particularly in free-space optical communications links. As a first step, we chose work in the eye-safe region around 1550 nm. However, the working spectral region can be allocated anywhere in the IR [22] and even the THz region [23] by a suitable choice of the local laser oscillation wavelength and the nonlinear crystal. We successfully transmitted a 2D QR code embedded in an IR laser beam and read it with a smartphone. Most image up-conversion research was made with targets illuminated in transmission mode and with objects or targets located in the objet focal plane of the up-converting system. Here, we conducted experiments in reflection mode with an object located farther than a focal length to the system. The procedure is shown in Figure 1.

Presently, free-space optical links were identified as the most attractive solution for last-mile wireless communications and in particular field-deployable transient links for military applications in the battlefield [24,25]. Robustness, low weight, low power consumption, low price, simplicity, and data security against eavesdropping are of course highly desirable characteristics for the battlefield. Based on a time-modulated eye-safe laser beam FSOC (free-space optical communications) links can be easily realized by combining an amplified telecom diode laser source, an electro-optic modulator, and a receiver based on an InGaAs p-i-n photodiode. On a point detector basis, the information can be sent via bit streams using OOK (on-off keying) as in standard fiber-optic communication systems. If no data encryption is used, even the weak scattering resulting in clean air (although there are always aerosols present that increase the scattered power significantly) can provide an eavesdropper placed out of the laser beam path with a sample of the bit stream. Even if the sample is very weak, an eavesdropping receiver based on a widespread InGaAs APD (avalanche photodiode) can do the job of recovering the bit stream as it is contained in the temporal structure of the transmitting laser and thus that of the scattered light. An example that illustrates the possibility of retrieving information from the weak scattering of an IR laser beam in air used for wind speed sensing and the benefits of using up-conversion can be found in another study [26]. However, when the information is contained in the spatial structure of the light, the scattering process destroys it and the eavesdropper cannot retrieve the transmitted data, even when using a camera rather than a photodiode. Although encryption enhances the security in the serial data transmission, one cannot discard a-priori knowledge by the eavesdropper of the encryption algorithm or the encryption key. Quantum encryption can always be used, but at the expense of increasing the link complexity. Thus, simple field deployable optical links can be made more secure though transmission of images than in binary sequential transmission of bit streams. Further, this is compatible with using an additional mathematical encryption algorithm in the 2D image of a QR code if desired.

Regarding data transmission speed, it is relatively easy at present to achieve around 40 Gb/s (limited by the speed of electronics) with standard off-the-shelf telecom components for fiber optics communications at 1550 nm in a free-space optical link based on beam on/off sequential data transmission (on-off keying). Contrary to the case of the eavesdropping system, rather than an APD, a p-i-n photodiode that requires no quenching can be used in the receiver due to the higher light level received, although APD bandwidths of 16 GHz have been recently reported [27].

A typical low cost B/W (black and white) Si camera with 10 × 10 µm pixels and 640 × 512 pixels operating at a frame rate around 50 fps (frames per second), can at most receive 16.38 Mb/s of binary data. Thus, the information capacity of a relatively low cost sequential system is presently superior to that of QR transmission with also low cost common equipment. Many field-deployable links can require not more than the 16.38 Mb/s. In this case, the main benefit of using up-conversion would be data protection. This could be increased inexpensively by using a grey scale rather than binary transmission. For instance, adding an 8-bit grey scale, the capacity would increase up to 4.19 Gb/s.

Rather than using a silicon camera combined with an up-conversion system, an InGaAs camera could be used in the eye-safe region. The main drawbacks are its much higher price, a typical one order of magnitude more in read noise (electrons/pixel) for TEC (thermoelectric cooler) cooled cameras and the typical 99% operative pixels due to the less mature fabrication technology of InGaAs FPA sensors as compared with the mature Si fabrication technology. Non-operative pixels are an important drawback for this application problem, as they would generate errors that could be accounted for by demanding more time dedicated to error-correction techniques, or by pixel grouping that would reduce the bit rate. Until recently InGaAs cameras have been of limited access due to ITAR regulations [28] and there is less availability than in Si cameras. However, despite these issues an enlightening comparative analysis can be made for transmission limits with 2D single-beam, single-wavelength modulation. This technique requires beam modulation in the transmitter.

Presently, the two main commercially available resources for 2D spatial modulation of the IR beam at the transmitter are transmission (amplitude) spatial light modulators (SLM) based on liquid crystals or ferroelectric pixels. Liquid crystal SLMs are inherently slower, but ferroelectric (expensive and of little availability) can operate typically with several megapixel frames at frame rates close to ≈1 kfps [29], and DMDs (digital mirror devices), a kind of MEMS (micro electric mechanical systems) with ≈4 megapixel frames at ≈15 kfps for binary modulation of the pixels (~ 60 Gb/s) [30]. According to the kind of modulator used in the transmitter, the physical situation is closer to using a target in transmission mode (SLM) or in reflection mode (DMD). Most of the previous work in image up-conversion has used targets in transmission mode. Here, we experiment in reflection mode, where faster DMD beam modulation speed can be presently achieved with commercial devices, although no significant changes in overall performance of the up-conversion system are expected in principle.

The limitations of direct image detection speed in the SWIR (1550 nm) using commercial InGaAs cameras are infrared are presently [31] 1700 fps at full frame resolution of 640 × 512, giving a maximum rate of 0.55 Gb/s (with a 99% pixel operability). Thus, present DMDs overflow their capacity. In case of InSb cameras for the SWIR/MWIR, the situation is quite similar, with 1000 fps at a full fame resolution of 640 × 512 [32]. However, a fast commercial Si camera (only black and white is needed), can reach 13 kfps (color camera) at a full resolution frame of 2048 × 1536 pixels [33]. Si image sensors’ speed limits are more diffuse and although not commercially available yet, 16 Mfps at a reasonable 256 × 256 resolution have been obtained in the lab, and there is present work towards the 1 Gb/s [34]. A parallel speed performance is not envisaged yet for IR image sensors.

Thus, for raising speed in IR imaging with an FPA final sensor, up-conversion combined with Si FPA image sensors seems to have no competitor. We recall that the CW SFM process is essentially instantaneous even at the Gb/s frame rate. Although not a theoretical limitation, the resolution achievable with a point-spread-function (PSF) of around 10 µm in a quite simple miniaturized up-conversion system and in a typical 1/2” format Si CCD sensor of 8 mm diagonal (6.4 × 4.8 mm) used in standard B/W cameras [35] is comparable to 640 × 512 pixels, although in view of the comments following Equation (3), little effort is required to bring it comparable to the 1280 × 1024 pixels resolution level. Thus, in terms of information capacity, SWIR, MWIR, and LWIR cameras overflow actual DMDs, while Si cameras overflow DMS capacity in binary transmission.

The CW intra-cavity image up-conversion technique is essentially instantaneous and it is therefore open to using high-speed Si cameras operating at 1000 fps or more at full resolution if required. In this sense, InGaAs or InSb IR cameras can reach high speed (frame rate) through pixel grouping (binning) to maintain a reasonable S/N, or by reading only regions of interest (RoI) within the full frame. Both speed-increasing techniques take place at the expense of resolution. Thus, the ultimate performance for data capacity is theoretically limited by the combination of frame rate and sensor resolution.

Due to the relatively recent renaissance of the field, there is much room for performance improvement. Specific effort to achieve QE ≈1 using known resources has not been the aim of the work in the field. However, 40% conversion efficiency in power has been reported under non-optimal conditions [21]. Rather, the effort has focused on solving FOV (field of view) and resolution issues. The room for high improvement in these characteristics is well supported by theory.

Another appealing advantage of image up-conversion to the VIS/NIR in many applications is the possibility of imaging at the single photon level in conjunction with an ICCD (intensified CCD camera) or Si-EMCCDs (electron multiplying CCDs). Such performance has already been demonstrated in the MWIR, with imaging at 0.2 photons/s per pixel [36]. This performance cannot be obtained with present IR image detectors, due to the lack of suitable photocathodes or EMCCDs not based on Si. There is one exception of little interest at 1550 nm with an image intensifier tube that uses an electron bombarded CCD instead of a phosphor screen. It is of very restricted access [28,37], with gating capability of only down to 50 ns, a 640 × 512 pixels frame, and 30 fps with a 98% pixel operability (blemish) characteristic of image intensifiers that use MCP (micro channel plates). ICCDs suffer from blemish giving less than 100% pixel operability and EMCCDs have hot and black pixels leading also to less than 100% pixel operability.

## 2. SFM Image Up-Conversion Background

**Figure 2:**
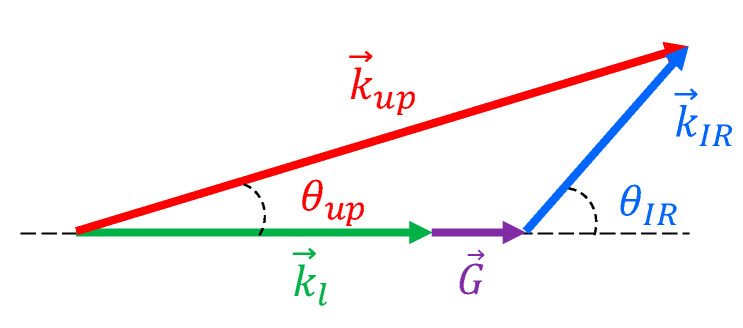
Sum-frequency mixing (SFM) image up-conversion process.

A plane-wave spatial Fourier component of spectral angle frequency ω_IR_ and wave-vector ${\vec{k}}_{\mathrm{IR}}$ in a 2D IR image—or, equivalently, an image ray in geometrical optics—is up-converted by SFM in a nonlinear crystal with a collimated laser beam of angle frequency ω_l_ and fixed wave-vector ${\vec{k}}_{l}$, to provide a Fourier component in a 2D up-converted image at a new angle frequency ω*_up_* = ω*_IR_* + ω*_l_*, and wave-vector ${\vec{k}}_{\mathrm{up}},$ where the relation ${\vec{k}}_{\mathrm{up}}-\left({\vec{k}}_{l}+{\vec{k}}_{\mathrm{IR}}\right)=\vec{G}$ needs to be fulfilled for efficient SFM through birefringent phase matching $\vec{G}\;\approx \;\vec{0}\;o$r for a reciprocal vector $\vec{G}$ of relevant weight (amplitude) contained within the Fourier expansion of the nonlinear spatial distribution of the nonlinear coefficient along the nonlinear crystal, a situation known as quasi-phase matching (QPM) [17]. Alternatively, the process can be viewed as an up-conversion of image rays in geometrical optics, depicted in Figure 2.

Although not restricted to, QPM is frequently achieved by creating a periodic spatial reversal in the spontaneous polarization orientation of ferroelectric domains in crystals like LiNbO_3_ (LN) or KTiOPO_4_ (KTP), known as PPLN or PPKTP. In each term, PP stands for periodically-poled. 

Typically, up-conversion takes place in the paraxial regime due to PM or QPM angle acceptance limitations and since in practice *n_up_* ≈ *n_IR_* and *G* << *k_l_*, where n stands for the refractive index, it can be derived from Figure 1 that the SFM process introduces an angle de-magnification factor given by:

(1) $$M_{up}=\frac{\theta_{IR}}{\theta_{up}}\approx \frac{\lambda_{u}\;}{\lambda_{IR}}$$

This factor is to be combined with other magnification factors due to the lenses in the optical system. It may be of interest to note that in spite of the change in wavelength and angle, an up-converted Fourier image component preserves the spatial frequency of the original IR Fourier component [38].

We point out that another parameter of interest is the FOV, which is ultimately limited by PM or QPM angle acceptance that is in general narrow. However, there are techniques to enlarge it, such as using a wider illumination spectrum, tangential phase matching, or broadening the QPM response with chirped structures or thermal gradients. In our application, a not too wide FOV is required. An estimation for the telescope configuration, without additional zoom or similar, is around 0.4 mrad (full cone angle), although it may be increased. However special effort must be done. Usual simple systems are around 50 mrad.

**Figure 3:**
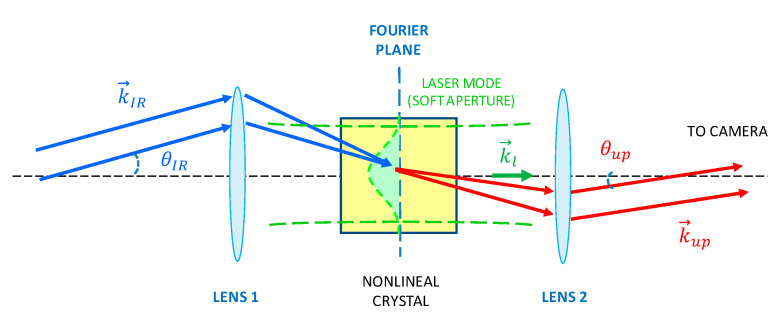
Telescope configuration for SFM image up-conversion.

Frequently, an image up-conversion system is built in a telescope configuration as shown in Figure 3, where the image focal plane of lens L1 coincides with the object focal plane of L2, thus creating a Fourier plane, where the center of the nonlinear crystal is placed. In case of intra-cavity up-conversion, the crystal is placed inside the cavity of a laser that provides the pump wave ${\vec{k}}_{1}$ for the SFM process, while the lenses are kept external to the laser cavity, and the SFM process takes place in an undepleted-pump regime. Most (if not all) reported image up-conversions locate the object and the FPA camera sensor in the object focal plane of L1 and image focal plane of L2, turning the telescope configuration into a 4-f Fourier processor setup.

Regarding conversion efficiency, in intra-cavity SFM up-conversion the nonlinear process takes place in the so-called undepleted pump regime that will be commented in more detail later. In that regime and for a collimated Gaussian pump beam, the relation between the intensity of an image point in the IR image and its up-converted point image intensity in a 4f Fourier processor setup can be well approximated using *F = 1* in the following equation [21]:

(2) $$I_{up}\left(x,y\right)=F\times \frac{16\pi^{2}\;d_{eff}^{2}\lambda_{IR}^{2}\;l^{2}}{n_{IR}n_{l}n_{up}c\varepsilon_{0}\lambda_{up}^{4}}\times \frac{f_{1}^{2}}{f_{2}^{2}}\times \frac{P_{l}}{\pi \omega^{2}}\times I_{IR}\left(x^{\prime },y^{\prime }\right)$$

Here, *I_u_ (x,y)* is the intensity of the up-converted image at the point *(x,y)* in a plane perpendicular to the propagation direction and in the image focal plane of lens L2 in Figure 3. *I_IR_ (x′,y′)* is the IR image intensity of a point located in the object focal plane of lens L1 in Figure 3 with coordinates *x′ = x/M_up_* and *y′ = y/M_up_*, accounting for the magnification factor *M_u_* defined in equation (2), *d_eff_* the effective second order nonlinear coefficient of the crystal, *l* the length of the nonlinear crystal, *c* and *ε_0_* the speed of light and dielectric constant in the vacuum, respectively, P_l_ the power of the Gaussian pump beam, ω its radius at 1/e^2^ intensity, *f_i_* the focal length of lens Li, and n_x_ and λ_x_ the refractive index inside the nonlinear crystal and the vacuum wavelength of wave *x* (*x* = IR, pump, up-converted). Because the object in a FSOC system is located at the transmitter (far away from the focal plane of L1), the telescope system can no longer be considered as a 4f setup, and the factor *F* is introduced to account for additional focusing or collecting optics present in a particular design. F is a constant within a design and will in general be *F* ≠ 1 for spatially modulated IR beams.

As seen, other parameters set, conversion efficiency increases quadratically with the value of the effective second-order nonlinear coefficient and linearly with the pump power density $\frac{P_{l}}{\pi \omega^{2}}$ in the nonlinear crystal. Because the circulating power inside a CW laser may typically be two orders of magnitude that at its output and nonlinear effective coefficients using QPM in poled ferroelectric crystals can reach typically around five times that achievable in frequently used crystals using birefringent phase-matching, placing a poled crystal inside the cavity of the laser that generates the pump wave, i.e., intra-cavity SFM, can notoriously enhance conversion efficiency, making image up-conversion presently more practical than in its early days. It is presently accepted that image up-conversion can essentially reach a theoretical QE ≈ 1 even at low IR incident photon fluxes. Strictly achieving QE = 1 in the sense that all incoming IR photons become up-converted requires some physical parameter to reach an infinite value. However, as an illustrative practical example for the case of beam (or wave-guided mode) up-conversion at room temperature, see for example [39], where 99% of the IR incoming photons become up-converted to their second harmonic. The theoretical principles underlying this performance rely on nonlinear optics theory and are valid for a beam, a wave-guided mode, or an image up-conversion, and extensible to SFM.

Regarding intra-cavity SFM, the work by Smith et al. [40,41] set the basis for achieving CW SFM with QE = 1 with the intra-cavity pump wave remaining undepleted. In essence, an IR photon needs only adding up to a pump photon to create an up-converted photon. Due to the large number of intra-cavity pump photons available with respect to the incoming IR photons, only a small depletion factor in the pump is required for QE = 1. However, the large number of pump photons traversing the intra-cavity nonlinear crystal that boosts conversion efficiency is indicated by Equation (2). In particular, when the intra-cavity pump intensity is very high, the favorable condition of a high *d_eff_* value condition can be somewhat relaxed and still achieve QE = 1 with non-poled birefringent crystals like KTP. The fact that no up-converted photons are created in the absence of IR photons sets the noiseless nature of the SFM process. However, a concern may remain regarding the spontaneous parametric down conversion of the pump beam creating photons at the IR wavelength not related to the IR signal, followed by a cascaded phase-matched SFM of the down-converted IR photons with the pump wave. This has also been demonstrated to generate a negligible amount of noise [42].

Equation (2) also reveals that in the undepleted regime the up-converted intensity of an image point is proportional to the corresponding object point in the original IR image, thus preserving a possible grey-scale in the process. Although much of the image up-conversion research has been made using binary amplitude targets in the lab, some examples of image up-conversion containing a grey-scale can be found in [22].

Concerning resolution, the highest resolution so far reported in SFM up-conversion imaging is around 100 lp/mm (line pairs per mm) for a target located in the object focal plane of L1 [43], corresponding to a PSF (FWHM) ≈10 µm using an input lens of focal length *f_1_* = 25 mm and an up-converted wavelength of 488 nm. This is not a fundamental limitation. The resolution limit is set by the diameter of the pump wave that acts as soft amplitude aperture in the Fourier plane of the system. Using laser illuminated targets and a Gaussian laser beam as the pump wave, the intensity PSF for SFM is also Gaussian and approximately set by:

(3) $$\mathrm{PSF}\left(r\right)\propto \;\exp-{\left\{\frac{\pi \;\omega \;r}{\lambda_{u}\;f_{1}}\right\}}^{2}$$

where *r* denotes the departure from the peak of the Gaussian and *ω* is the radius of the collimated pump laser Gaussian beam at 1/e^2^ intensity in the nonlinear crystal, *f*_1_ the focal length of the input lens to the system (L1 in Figure 3), and $\lambda_{u}$ the wavelength of the up-converted image. For instance, a 1 mm radius pump beam and a value *f*_1_ = 10 mm (possible with a miniaturized system architecture such as that described in [44]), leads to a 3% MTF cut-off spatial frequency of ≈360 lp/mm, and a Gaussian FWHM PSF radius ≈78 µm (~10 µm at 1/e^2^ radius) for an up-converted wavelength of 631 nm.

Recently, it has been shown that the image up-conversion process provides a natural mechanism for fast electro-optic image gating based on transient enable/frustration of the up-conversion process, which allows for range-gated systems in the IR in combination with an EMCCD camera [38]. It should be clear that extension spectral allocations different from 1550 nm are straightforward by selecting a different local oscillator laser wavelength combined with a suitable nonlinear crystal.

## 3. Experimental Setup

**Figure 4:**
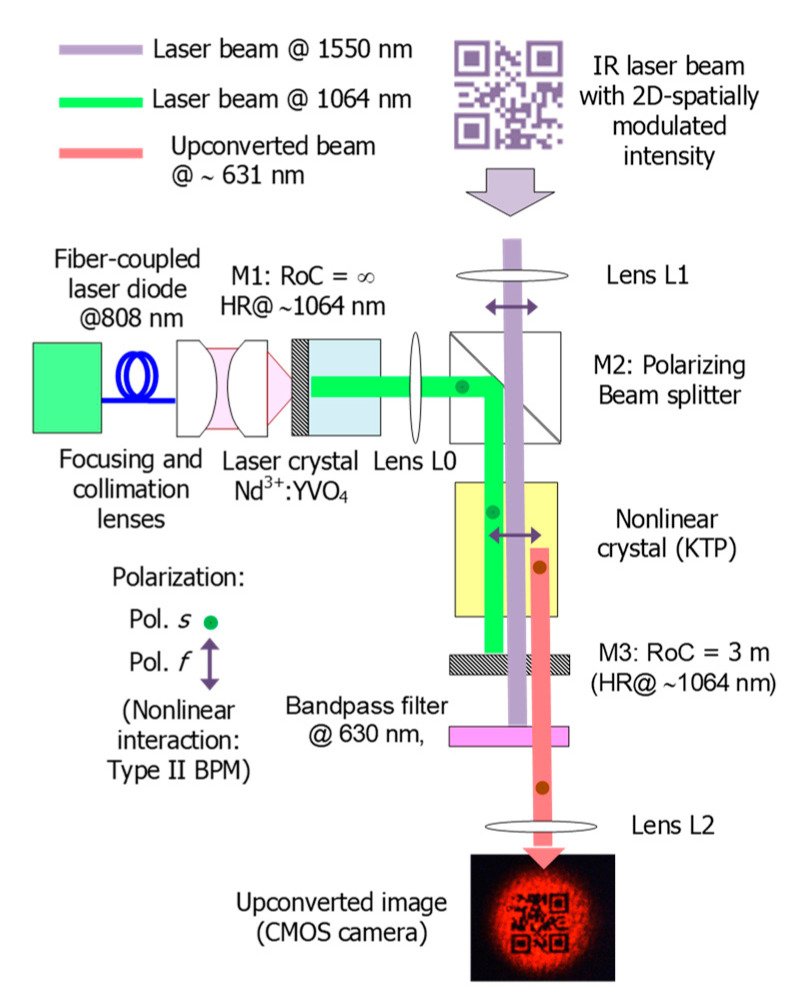
Experimental setup of the image up-conversion system proposed for sensing.

The setup of the image up-conversion system proposed in this work is shown in Figure 4. The system consists of an 808 nm-diode-pumped Nd^3+^:YVO_4_ solid-state laser, inside of which a nonlinear crystal mixes the CW laser oscillation at 1064 nm, acting as the auxiliary pump laser, with the IR beam carrying 2D spatially-modulated information. The IR beam results from illumination of a patterned surface (2D data codes) by using a collimated laser at 1550 nm to collect such information remotely according to the arrangement shown in Figure 1. Thus, the original IR information is shifted to the visible spectrum centered at 631 nm by SFM in a single-pass nonlinear interaction inside the laser cavity (intra-cavity conversion). 

The solid-state laser consists of a linear cavity folded at a right angle to facilitate the coupling of the original 2D information beam inside for up-conversion. The cavity is delimited by a flat input mirror (M1) deposited on the Nd^3+^:YVO_4_ laser crystal presenting high reflectivity (HR, typically with *R* ≥ 99.5% for commercial standard composites) and high transmission (HT) at 808 nm on the outer cavity side, and by a nearly flat mirror (M3) of 3 m radius of curvature acting as the output coupler. The mirror M3 is HR at 1064 nm and HT in the visible and around 1550 nm, and has spherical geometry to keep the cavity stable and to provide precise control of the laser mode size. In addition, the radius of M3 is long enough to be considered a flat surface and thus avoids distortion of the up-converted image at the cavity output. A polarizing beam splitter (PBS) is used as the folding mirror M2. The PBS is HR at 1064 nm on the cavity side for the linearly polarized laser oscillation, according to the vertical axis (perpendicular to the figure plane). The outer face is HT at 1550 nm for horizontal polarized beams (contained in the figure plane) so that eye-safe beams carrying 2D information are coupled inside the cavity to be mixed with the laser oscillation. As a laser crystal, a 4 mm long and 3 × 3 mm^2^ cross-section Nd^3+^:YVO_4_ crystal with 3% at. Nd^3+^ doping is used, presenting flat-parallel facets with AR coating at 1064 nm on the cavity side. The laser crystal is end-pumped by a fiber-pigtailed diode laser at 808 nm. Despite that the Nd^3+^:YVO_4_ crystal is a-cut to provide linearly polarized laser oscillation, the crystal must be oriented so that the laser polarization is parallel to both the PBS constraint and the slow axis of the nonlinear crystal, as described next. As a mixer, a bulk crystal of potassium titanyl phosphate (KTP) is used intra-cavity for single-pass SFM through birefringence phase matching (BPM). The KTP is an 8 mm long 6 × 6 mm^2^ in cross-section biaxial crystal cut at θ = 55° and ϕ = 0° for critical type-II BPM of the targeted SFM process: 1550 nm + 1064 nm → 631 nm. In this way, the nonlinear interaction is depicted in Figure 4 following the nomenclature associated to biaxial crystals. Then, the KTP crystal is oriented so that the 1550 nm beam is linearly polarized according to the fast axis *f* (also called ordinary component) of the KTP crystal while the 1064 nm beam is linearly polarized according to the slow axis *s* (extraordinary component), so that the up-converted beam at 631 nm is linearly polarized with the fast component. For the targeted process, the KTP crystal exhibits an effective nonlinear coefficient around 3 pm/V and its facets are both coated for AR at 1064 nm.

**Figure 5:**
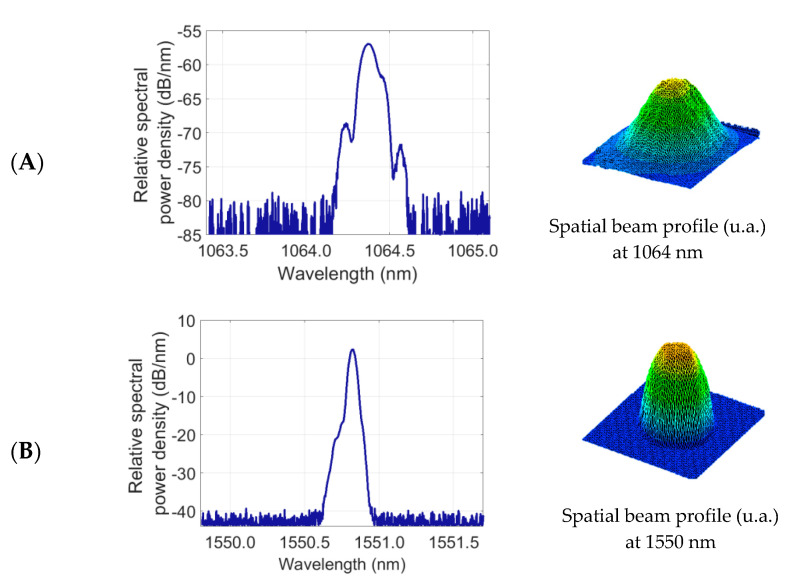
Spectra and spatial profiles of the interacting waves: (**A**) 1064 nm beam at the cavity output and (**B**) 1550 nm beam before 2D spatial modulation.

A plano-convex lens L0 with *f*_0_ = 50 mm focal length is placed between the Nd^3+^:YVO_4_ crystal and the PBS in order to control the size of the fundamental laser mode in the laser crystal. In this way, optimal overlapping with the pumping mode at 808 nm is achieved and, as a consequence, the lasing threshold can decrease. The lens L0 is AR coated at 1064 nm to avoid losses leading to a penalty of the intra-cavity power density. This lens allows fine adjustment of the laser beam size to the KTP cross-section while providing a nearly collimated beam to prevent distortion from up-converted patterns. The spectra and spatial profiles of the interacting beams at 1064 nm and at 1550 nm (before spatial modulation) are shown in Figure 5. The laser mode presents a Gaussian profile helping to avoid distortion and loss of resolution of the up-converted pattern. The proposed image up-conversion system is arranged following a telescope configuration through the lenses L1 and L2. L1 is a plano-convex lens with a focal length of *f*_1_ = 125 mm used for collecting the collimated beam resulting from IR illumination of targeted surfaces and the subsequent focusing onto the KTP crystal (Fourier plane). L2 is also a plano-convex lens with a focal length of *f*_2_ = 125 mm used for the collimation of the up-converted beam prior focusing on the CMOS (complementary metal-oxide-semiconductor) sensor and thus for the pattern formation and subsequent decoding. Finally, a bandpass filter at 630 nm (10 nm FWHM) is placed between the output coupler M3 and the lens L2 to block the propagation of the remaining laser output and the unabsorbed 808 nm pump to the CMOS camera.

## 4. Results and Discussion

**Figure 6:**
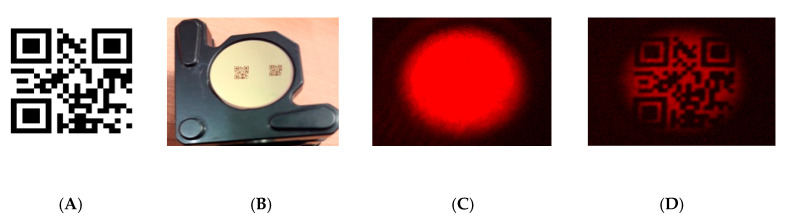
(**A**) QR data pattern used for spatial modulation. (**B**) Reflecting surface with printed 2D data. Up-converted beam images: (**C**) unmodulated and (**D**) modulated and read out.

The experimental setup described in Figure 4 is used to show the potential of up-conversion sensing for retrieving 2D information remotely through standard CMOS cameras in active image systems operating in the eye-safe wavelength region. For this aim, a DFB (distributed feedback) laser at 1550 nm is used for illumination of targeted surfaces with printed information based on 2D matrix codes. The laser beam is collimated and presents a Gaussian spatial profile with a spot size of 5-mm in diameter before spatial modulation, as shown in Figure 5B. The reflecting patterns are located at a distance of about 1 meter from the input of the image up-conversion system. The patterns are arranged in a 21 × 21-bit matrix resulting from the coding of a website address, according to the QR standardized format, as shown in Figure 6A. The spatial modulation results when the laser beam illuminates the reflecting patterns that consist of masks with the 2D data matrix deposited on a reflecting surface (gold mirror with 98.5% reflectance at 1550 nm for p-polarization at approximately 0° angle of incidence). The masks are made of a 0.18-mm-thick acetate film (Kodak film ARD7) with data printed by a photo-plotter (1625 dpi resolution), as shown in Figure 6B. The masks employed are identical to those used in transmission in a previous setup focused on the demonstration up-conversion of spatially modulated beams in FSOC applications [1]. Then, the beam carrying 2D information at 1550 nm is polarization coupled to the laser cavity for mixing with the laser oscillation at 1064 nm in the KTP crystal. The spatial profile of the up-converted beam at 631 nm also attains a Gaussian profile in absence of modulation, as shown in Figure 6C. The up-converted 2D pattern is formed onto the sensor surface of the CMOS camera, as shown in Figure 6D, and has a resolution that is high enough to be successfully decoded.

The resolution of the up-conversion system, which is described by the point spread function (PSF) of the system in terms of Fourier Optics, is limited by the size of the laser mode [45,46]. Then, the laser mode acts as a soft amplitude aperture in the Fourier plane by filtering out the higher frequency spatial components from the original pattern. The Gaussian profile of the laser mode makes the S/N achieved at the receiver side lower as the bit position is farther away from the image center. The use of a shorter focal length for L1 would allow the increase of the resolution of the up-conversion system, as described in (3). In the same sense, there also lies the importance of the enlargement of the laser mode size to the cross-section of the non-linear crystal. According to the proposed configuration, the location and the focal length of the lens L0 determine the size and the collimation degree of the laser mode. Thus, shorter focal lengths of L0 would allow the increase of the laser mode size in the Fourier plane. As a result, increasing the pumping power at 808 nm would compensate the reduction of the laser power density. However, this option would make accessibility inside the cavity difficult for fine adjustment and alignment of optical elements and for the experimental characterization. In our setup, the use of a lens L1 with a short focal length may restrict the placement of the PBS and, as a consequence, cavity size.

**Figure 7:**
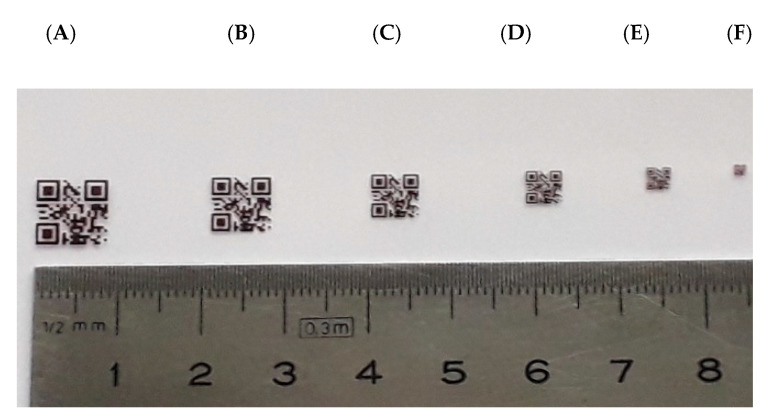
Masks used for 2D data spatial modulation of the eye-safe beams: (**A**) 8 mm × 8 mm, (**B**) 7 mm × 7 mm, (**C**) 5.5 mm × 5.5 mm, (**D**) 4 mm × 4 mm, (**E**) 2.5 mm × 2.5 mm and (**F**) 1.25 mm × 1.25 mm.

The spatial resolution of the up-conversion system is evaluated by illumination of reflecting targets built with the patterns shown in Figure 7. All of the 2D patterns contain the same data but have different sizes of code and bit. The bit size is associated to the corresponding element of the USAF-1951 standard for resolution testing. In this way, the 2D pattern size ranges from 8.0 mm × 8.0 mm for the mask A, to 1.25 mm × 1.25 mm for the mask F; and the corresponding bit size varies between 800 μm × 800 μm for the mask A (equivalent to the group 1, element 3 of the USAF-1951 standard), and 60 μm × 60 μm for the mask F (with equivalence to the group 3, element 1 of the mentioned standard).

**Figure 8:**
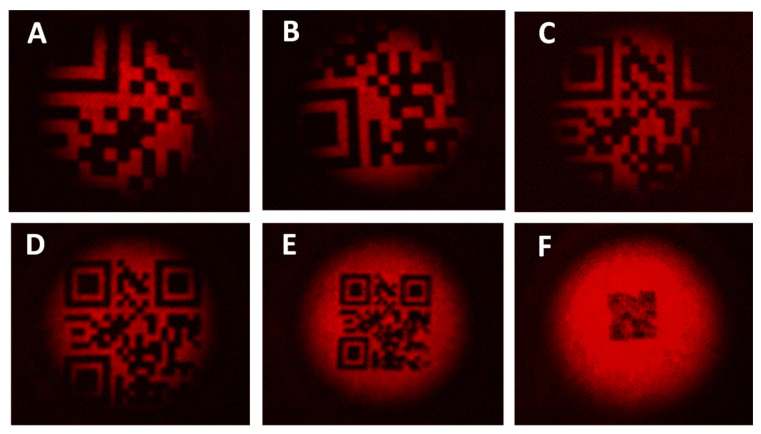
Up-converted QR codes at 631 nm corresponding to the 2D data masks shown in Figure 7: (**A**) 8 mm × 8 mm, (**B**) 7 mm × 7 mm, (**C**) 5.5 mm × 5.5 mm, (**D**) 4 mm × 4 mm, (**E**) 2.5 mm × 2.5 mm and (**F**) 1.25 mm × 1.25 mm.

The up-converted QR-code patterns at 631 nm are shown in Figure 8 when the reflecting targets (A-F) are illuminated with the 5-mm-diameter beam at 1550 nm. Despite there being no resolution limitation or contrast loss in patterns shown in Figure 8A and 8B, these up-converted QR-codes cannot be decoded since relevant information associated to the positioning and synchronization is missed. In contrast, the up-converted pattern represented in Figure 8C preserves all the bits associated to the encoded information and synchronization but the loss of positioning references prevents from being decoded too.

The up-converted QR-codes corresponding to the patterns D and E are entirely found in the FOV of the system. Both codes show resolution levels of ~190 μm × 190 μm (group 1, element 3) and ~120 μm × 120 μm (group 2, element 1), respectively, and allow decoding and information access correctly. On the contrary, the up-converted QR-code associated to the reflecting pattern F exhibits a significant degradation in terms of resolution. This is because the mask F has a bit size of 60 μm × 60 μm, which is much smaller than the theoretical resolution limit set at 100 μm × 100 μm by the PSF for a focal length of *f*_1_ = 125 mm and a laser beam diameter of ~ 700 μm in the middle of the KTP crystal.

The formation of the up-converted 2D pattern undergoes the resizing of the original IR pattern in the image plane of the telescopic system due to two sources of magnification. The first contribution is associated to the type-II SMF nonlinear interaction as a result of the fulfillment of the momentum conservation principle. In particular, this effect leads to the angular de-magnification, which is proportional to the factor ~ (λ_631_/λ_1550_) ≈ 0.41 given by the up-converted wavelength to the eye-safe wavelength ratio [47]. The second contribution is determined by the ratio of focal lengths of lenses L1 and L2 of the telescopic configuration (*f*_1_/*f*_2_) and the factor *F* (Equation (2)), which accounts for additional focusing optics elements used in the system. Apart from that, the amount of information up-converted per frame is also limited by the FOV, which is therefore determined by the illumination beam size, the angular acceptance, and optics of the telescopic configuration. In this context, several techniques have been proposed to date [48,49,50,51] in order to enhance the FOV, but the implementation of such improvements goes beyond the scope of this work.

For the laser cavity conditions described in Section 3, the laser oscillates along the KTP crystal with a nearly collimated mode of ~700 μm in diameter. When the Nd^3+^:YVO_4_ crystal is pumped with 1 W at 808 nm, the up-converted image begins to be dimly displayed on the CMOS camera for a power level of about 200 μW at 1550 nm. Although the up-conversion efficiency achieved is far from predicted in theory [21,40], it is not a concern in this work since up-converted power levels obtained are high enough to meet the saturation threshold of the CMOS camera. If higher efficiency were required, the use of PPLN or PPLT crystals would offer a higher effective coefficient (~ 15 pm/V) than that exhibited by the bulk nonlinear crystal. Nevertheless, current manufacturing techniques cannot achieve crystal cross-sections wider and thicker than 1 mm, simultaneously, when poling periods required for QPM are under approximately 20 μm. Hence, the choice of the phase matching technique, or rather the kind of nonlinear crystal (bulk versus periodically poled technology), involves a trade-off between efficiency and resolution. In the case of bulk crystals, increasing the intra-cavity power of the pump laser can easily compensate the up-conversion efficiency.

## 5. Conclusions

Together with some preliminary results recently presented [1], this work introduces up-conversion imaging in the context of optical communications, particularly in FSOC systems. As a first demonstration, a 21 × 21-bit matrix 2D QR binary code embedded in an 1550 nm IR laser beam is transmitted in the laboratory and successfully received and recognized by a smartphone’s Si camera and standard QR recognition software. Compared to an actual FSOC based on sequential (1D) transmission of bit streams by on/off beam modulation, coding the information in the 2D structure of the beam provides increased security against eavesdropping by beam scattering in air. Working in the IR eye-safe region allows for increased eye-safety in laser beams and avoids rapid visual detection of the transmitter location and the line to the receiver. An FPA-based camera is the most convenient way of sensing the 2D structure within a laser beam. However, due to IR FPA-sensors limitations up-conversion imaging to the VIS/NIR is in general lower cost and outperforms IR cameras in transmission speed. We have shown that it is presently the only technology than can compete and even surpass the ~40 Gb/s easily achievable with a sequential FSOC on a single-wavelength laser basis, i.e., without using wavelength multiplexing in any of the 1D or 2D systems.

Here, we used a KTP nonlinear crystal placed inside the cavity of a Nd^3+^:YVO_4_ laser oscillating at 1064 nm, to mix a spatially modulated (QR code) 1550 laser beam with the 1064 nm to obtain a 631 nm up-convert SFM image of the QR code. In our experimental conditions, we differentiate size of up to 100 μm × 100 μm. However, this is not a limitation and future attempts will reasonably achieve the 10 μm × 10 μm and even smaller sizes. By changing the laser crystal and the nonlinear crystal, a similar system may operate in the MWIR or LWIR, if desired. 

The system can be miniaturized down to a quasi-monolithic robust architecture around 4 cm^3^ and built at a low cost with standard commercial components, resulting lightweight, and favoring field-deployable IR eye-safe links, although it is easily extensible to the MWIR and LWIR spectral regions.

Presently, the fastest commercial beam modulators are DMDs, capable of generating four megapixel binary images at frame rates of ~10 kfps binary images upon reflection of a beam. Despite their high price and cooling, InGaAs and InSb cameras can provide no more than 1 kfps at full resolutions of ~1 megapixel at present. Faster speeds are possible via pixel grouping or RoI reading, at the expense of a severe loss of bits in the frame (resolution). Thus, present DMS overflows the capabilities of InGaAs, InSb, or HgCdTe cameras. On the contrary, fast Si cameras overflow DMD. In addition, since the SFM process is noiseless and can achieve QE ~1, the S/N ratio in the system is set by the readout noise. Since Si cameras have typical read out noise values around 0.5-5 electrons/pixel and cooled InGaAs and InSb around 50 electrons/pixel up-conversion, detection with a Si camera can outperform direct IR detection in S/N ratio. Because intra-cavity up-conversion essentially preserves the grey scale in an image, up-conversion systems can support multilevel bits. At larger distances, using structured laser beams may help to keep good data rates with moderate apertures in the system due to resolution issues. 

## References

[1] Torregrosa A.J., Maestre H., Rico M.L., Karamehmedović E., Capmany J. Up-conversion of eye-safe beams carrying 2D spatially-modulated information for detection with Si-FPA cameras in FSO applications. Proceedings of the 8th International Conference on Fiber Optics in Access Networks (FOAN 2019).

[2] Tofail S.A.M., Mani A., Bauer J., Silien C. (2018). In Situ, Real-Time Infrared (IR) Imaging for Metrology in Advanced Manufacturing. Adv. Eng. Mater..

[3] Choe G., Park J., Tai Y.W., Kweon I.S. (2017). Refining Geometry from Depth Sensors using IR Shading Images. Int. J. Comput. Vis..

[4] Telenkov S.A., Smithies D.J., Goodman D.M., Tanenbaum B.S., Nelson J.S., Milner T.E. (1998). Infrared imaging of in vivo microvasculature following pulsed laser irradiation. J. Biomed. Opt..

[5] Van de Asperen Boer J.R.J. (1968). Infrared Reflectography: A Method for the Examination of Paintings. Appl. Opt..

[6] Redman B.J., van der Laan J.D., Westlake K.R., Segal J.W., La Casse C.F., Sanchez A.L., Wright J.B. (2019). Measuring resolution degradation of long-wavelength infrared imagery in fog. Opt. Eng..

[7] Uthe E.E. (1981). Lidar evaluation of smoke and dust clouds. Appl. Opt..

[8] Case J.R., Young M.A., Dreau D., Trammell S.R. (2015). Noninvasive enhanced mid-IR imaging of breast cancer development in vivo. J. Biomed. Opt..

[9] Lemoff B.E., Martin R.B., Sluch M., Kafka K.M., McCormick W., Ice R. Long-Range Night/Day Human Identification using Active-SWIR Imaging. Proceedings of the SPIE 8704, Conference on Infrared Technology and Applications XXXIX.

[10] Robert P., Bertrand D., Devaux M.F., Sire A. (1992). Identification of Chemical Constituents by Multivariate Near-Infrared Spectral Imaging. Anal. Chem..

[11] Everitt J.H., Escobar D.E., Villarreal R., Noriega J.R., Davis M.R. (1991). Airborne video systems for agricultural assessment. Remote Sensing of Environment. Appl. Eng. Agric..

[12] Ceccarelli S., Guarneri M., Ferri de Collibus M., Francucci M., Ciaffi M., Danielis A. (2018). Laser Scanners for High-Quality 3D and IR Imaging in Cultural Heritage Monitoring and Documentation. J. Imaging.

[13] Meriaudeau F. Infrared imaging and machine vision. Proceedings of the SPIE 6503, Machine Vision Applications in Industrial Inspection XV.

[14] Killinger D.K., Menyuk N. (1987). Laser Remote Sensing of the Atmosphere. Science.

[15] Kleiman D.A., Boyd G.D. (1969). Infrared Detection by Optical Mixing. J. Appl. Phys..

[16] Franken P.A., Hill A.E., Peters C.W., Weinreich G. (1961). Generation of Optical Harmonics. Phys. Rev. Lett..

[17] Armstrong J.A., Bloembergen N., Ducuing J., Pershan P.S. (1962). Interactions between Light Waves in a Nonlinear Dielectric. Phys. Rev..

[18] Midwinter J.E., Warner J. (1967). Up—Conversion of Near Infrared to Visible Radiation in Lithium—meta—Niobate. J. Appl. Phys..

[19] Pedersen R.L., Hot D., Li Z. (2018). Comparison of an InSb Detector and Upconversion Detector for Infrared Polarization Spectroscopy. Appl. Spectrosc..

[20] Midwinter J.E. (1968). Image conversion from 1.6 µm to the visible in lithium niobate. Appl. Phys. Lett..

[21] Pedersen C., Karamehmedović E., Dam J.S., Tidemand-Lichtenberg P. (2009). Enhanced 2D image upconversion using solid-state lasers. Opt. Express.

[22] Barh A., Rodrigo P.J., Meng L., Pedersen C., Tidemand-Lichtenberg P. (2019). Parametric upconversion imaging and its applications. Adv. Opt. Photon..

[23] Fan S., Qi F., Notake T., Nawata K., Takida Y., Matsukawa T., Minamide H. (2015). Diffraction-limited real-time terahertz imaging by optical frequency up-conversion in a DAST crystal. Opt. Express.

[24] Juarez J.C., Dwivedi A., Hammons A.R., Jones S.D., Weerackody V., Nichols R.A. (2006). Free-Space Optical Communications for Next-generation Military Networks. IEEE Commun. Manag..

[25] Harris A., Al-Akkoumi M., Al-Akkoumi M.K., Sluss J. (2012). Mobile free-space optical communications: A feasibility study of various battlefield scenarios. Proc. SPIE.

[26] Shangguan M., Xia H., Wang C., Qiu J., Shentu G., Zhang Q., Dou X., Pan J.-W. (2016). All-fiber upconversion high spectral resolution wind lidar using a Fabry-Perot interferometer. Opt. Express.

[27] Zhao H.-Y., Jones A.H., Chao R.L., Ahmad Z., Campbell J.C., Shi J.-W., , (2019). High-Speed Avalanche Photodiodes with Wide Dynamic Range Performance. IEEE J. Lightwave Tech..

[28] International Traffic in Arms Regulations—ITAR. https://research.mit.edu/integrity-and-compliance/export-control/information-documents/export-control-regulations.

[29] Boulder Nonlinear Systems, Spatial Light Modulators XY-SERIES. http://bnonlinear.com/pdf/XYSeriesDS0909.pdf.

[30] Texas Instruments DMD DLP900. https://www.ti.com/lit/ds/symlink/dlp9000.pdf.

[31] High Image Resolution InGaAs Camera. Cheetah 640 Series. Xenics Infrared Solutions. https://www.xenics.com/short-wave-infrared-imagers/cheetah-series/.

[32] General Purpose Thermography Cameras. Spark IR-SERIES. TELOPS. https://www.telops.com/products/thermography-cameras.

[33] High Resolution and High Speed Cameras. i-SPEED 7 Series. IX-Cameras. https://www.ix-cameras.com/7-Series/index.php.

[34] Etoh T.G., Dao V.T.S., Shimonomura K., Charbon E., Zhang C., Kamakura Y., Matsuoka T. Toward 1Gfps: Evolution of Ultra-high-speed Image Sensors -ISIS, BSI, Multi-Collection Gates, and 3D-stacking. Proceedings of the 2014 IEEE International Electron Devices Meeting.

[35] 1/2-Inch Sony-ICX415 CCD-Sensor-Based Camera. https://www.edmundoptics.com.sg/p/guppy-f-046-firewirea-frac12quot-ccd-monochrome-camera/15787/.

[36] Dam J.S., Tidemand-Lichtenberg P., Pedersen C. (2012). Room-temperature mid-infrared single-photon spectral imaging. Nat. Photon..

[37] Gated Short Wave Infrared (SWIR) Camera System. Intevac. https://www.intevac.com/intevacphotonics/livar-506/.

[38] Capmany J., Torregrosa A.J., Maestre H. (2020). Intracavity image upconversion system with fast and flexible electro-optic image gating based on polarization-frustrated phase-matching for range-gated applications. Opt. Express.

[39] Parameswaran K.R., Kurz J.R., Roussev R.V., Fejer M.M. (2002). Observation of 99% pump depletion in single-pass second-harmonic generation in a periodically poled lithium niobate waveguide. Opt. Lett..

[40] Smith R.G. (1970). Theory of Intra-Cavity Optical Second-Harmonic Generation. IEEE J. Quantum Electron..

[41] Geusic J.E., Levinstein H.J., Singh S., Smith R.G., Van Utert L.G. (1968). Continuous 0.532 μm solid-state source using Ba_2_NaNb_5_O_15_. Appl. Phys. Lett..

[42] Tang C.L. (1969). Spontaneous Emission in the Frequency Up-Conversion Process in Nonlinear Optics. Phys. Rev..

[43] Dam J.S., Pedersen C., Tidemand-Lichtenberg P. (2010). High-resolution two-dimensional image upconversion of incoherent light. Opt. Lett..

[44] Torregrosa A.J., Maestre H., Rico M.L., Capmany J. (2018). Compact self-illuminated image upconversion system based on intracavity second-harmonic generation. Opt. Lett..

[45] Goodman J.W. (2005). Introduction to Fourier Optics.

[46] Karamehmedović E., Pedersen C., Jensen O.B., Tidemand-Lichtenberg P. (2009). Nonlinear beam clean-up using resonantly enhanced sum-frequency mixing. Appl. Phys. B.

[47] Midwinter J.E. (1968). Parametric infrared image converters. IEEE J. Quantum Electron..

[48] Demur R., Garioud R., Grisard A., Lallier E., Leviandier L., Morvan L., Treps N., Fabre C. (2018). Near-infrared to visible upconversion imaging using a broadband pump laser. Opt. Express.

[49] Maestre H., Torregrosa A.J., Capmany J. (2016). IR image upconversion under dual-wavelength laser illumination. IEEE Photonics J..

[50] Torregrosa A.J., Maestre H., Capmany J. (2015). Intra-cavity upconversion to 631 nm of image illuminated by an eye-safe ASE source at 1550 nm. Opt. Lett..

[51] Maestre H., Torregrosa A.J., Fernandez-Pousa C.R., Capmany J. (2018). IR-to-visible image upconverter under nonlinear crystal thermal gradient operation. Opt. Express.

